# First-Line Chemotherapy Versus CDK4/6 Inhibitors in HR-Positive, HER2-Negative Breast Cancer with Liver Metastases: A Multicenter Real-World Data

**DOI:** 10.3390/jcm14134568

**Published:** 2025-06-27

**Authors:** Mehmet Cem Fidan, Ahmet Emin Öztürk, Okan Aydın, Goncagül Akdağ, Ezgi Türkoğlu, Rumeysa Çolak, Nargiz Majidova, Tanju Kapağan, Murad Guliyev, Emir Çelik, Hatice Odabaş, Mesut Yılmaz, İbrahim Vedat Bayoğlu, Nilüfer Bulut, Nebi Serkan Demirci, Özkan Alan

**Affiliations:** 1 Division of Medical Oncology, Department of Internal Medicine, Cerrahpaşa Faculty of Medicine, Istanbul University-Cerrahpaşa, Istanbul 34098, Türkiye; drmuradguliyev@gmail.com (M.G.); drserkannebi@yahoo.com (N.S.D.); ozkan.alan@hotmail.com (Ö.A.); 2 Department of Medical Oncology, University of Health Science, Prof. Dr. Cemil Tascıoglu City Hospital, Istanbul 34360, Türkiye; draeozturk@gmail.com (A.E.Ö.); drokan29@gmail.com (O.A.); emircelikk@gmail.com (E.Ç.); 3 Department of Medical Oncology, Kartal Dr. Lütfi Kirdar City Hospital, Health Science University, Istanbul 34865, Türkiye; akdaggoncagul@gmail.com (G.A.); ezgiturk_90@hotmail.com (E.T.); odabashatice@yahoo.com (H.O.); 4 Department of Medical Oncology, University of Health Science, Bakirkoy Dr. Sadi Konuk Training and Research Hospital, Istanbul 34147, Türkiye; colak.rmys@gmail.com (R.Ç.); mesutyilmaz12@yahoo.com (M.Y.); 5 Division of Medical Oncology, Department of Internal Medicine, Marmara University School of Medicine, Istanbul 34899, Türkiye; nergiz.mecidova1991@gmail.com (N.M.); drvebay@gmail.com (İ.V.B.); 6 Department of Medical Oncology, Basaksehir Cam and Sakura City Hospıtal, Istanbul 34480, Türkiye; tanjukapagan2016@gmail.com (T.K.); nilufer.bulut@sbu.edu.tr (N.B.)

**Keywords:** CDK4/6 inhibitors, chemotherapy, endocrine resistance, HR-positive, HER2-negative breast cancer, liver metastasis, overall survival, palbociclib, progression-free survival, ribociclib

## Abstract

**Background**: Hormone receptor (HR)-positive, human epidermal growth factor receptor 2 (HER2)-negative metastatic breast cancer frequently involves liver metastases, which are linked to poor outcomes. The optimal first-line treatment strategy in this subgroup remains unclear. **Methods**: This multicenter, retrospective study evaluated 121 patients with HR-positive, HER2-negative breast cancer and liver metastasis who had not received prior systemic chemotherapy or cyclin-dependent kinase (CDK) 4/6 inhibitors (e.g., palbociclib, ribociclib). Patients were classified based on their initial treatment during the liver metastatic phase (CDK4/6 inhibitors or chemotherapy). Clinical characteristics, treatment patterns, and survival outcomes were assessed. Cox regression analysis identified independent prognostic factors. **Results**: The median age was 53 years; 62% were postmenopausal. Chemotherapy was administered to 36.4%, and CDK4/6 inhibitors to 63.6% of patients. Most cases (59.5%) were recurrent disease. Baseline characteristics were comparable, except for the use of local liver-directed therapies. Progression-free survival favored CDK4/6 inhibitors (10.9 vs. 4.8 months; *p* < 0.01), while overall survival favored chemotherapy (42.2 vs. 25.9 months; *p* = 0.042). In multivariate analysis, initial treatment modality, local liver-directed therapy, hormonal status, and the size of the largest liver lesion were independent predictors of survival. **Conclusions**: In patients with HR-positive, HER2-negative breast cancer and liver metastasis, first-line chemotherapy was associated with improved overall survival compared to CDK4/6 inhibitors. Local liver-directed therapies contributed to longer survival, while larger liver lesions and endocrine-resistant disease correlated with worse outcomes. These findings support considering chemotherapy as first-line treatment in selected patients with aggressive liver metastatic disease.

## Simple Summary

The presence of liver metastases in hormone receptor-positive and HER2-negative breast cancer is generally associated with poor prognosis and decreased survival. It is not clear whether CDK4/6 inhibitors are superior to other treatment options for this patient group. In our study, we evaluated the outcomes of patients who underwent chemotherapy or CDK4/6 inhibitors as first-line treatment with liver metastatic HR+/HER2- breast cancer. Our findings show that CDK4/6 inhibitors extended disease control by prolonging the progression-free interval, but chemotherapy was associated with increased overall survival. Moreover, we observed that survival durations decreased in individuals with hormone-resistant, larger liver metastatic lesions, whereas liver-directed local therapy was associated with improved survival. Our findings indicate that chemotherapy could serve as a first-line therapeutic option for certain individuals with aggressive liver metastases (e.g., hormone-resistant disease, large liver metastases).

## 1. Introduction

According to GLOBOCAN statistics, approximately 2.3 million new cases of breast cancer were reported in 2022, with an estimated 670,000 deaths attributed to the disease. Breast cancer (BC) is the most diagnosed malignancy among women, accounting for over 25% of all cases, and remains one of the leading causes of cancer-related mortality worldwide [1].

The hormone receptor (HR)-positive, human epidermal growth factor 2 (HER2)-negative (HR+/HER2-) subgroup accounts for 59% to 73% of breast cancer cases [2]. The 5-year survival rate for patients with metastatic breast cancer (mBC) is 24% to 39% [2]. At the time of diagnosis, 5% to 8% of breast tumors have distant metastases; however, approximately 25% to 40% of individuals diagnosed at earlier stages later develop metastatic disease despite advances in treatment [3].

The liver, which is one of the most common metastasis sites in adenocarcinomas [4], is one of the important metastasis sites due to its critical functions such as metabolism, detoxification, and protein synthesis. The presence of liver metastasis is independently associated with poor survival outcomes of primary cancer types [5]. The presence of liver metastases is generally associated with poor survival outcomes in patients with advanced HR-positive breast cancer, as in all breast cancer subgroups [6,7].

There are multiple clinicopathological and molecular factors that have been considered prognostic and predictive markers that may influence the clinician’s treatment decision in this patient population, other than the metastatic site. These include higher Ki67 levels, low or no progesterone receptor levels, HER2 status (zero or low), endocrine-resistant disease, and recurrent metastatic disease status, which are linked to more aggressive disease, resistance to hormone therapy, and worse survival rates [8,9,10,11,12,13].

Cyclin-dependent kinase (CDK) 4/6 inhibitors in combination with endocrine therapy (ET) have become the standard first-line treatment for most patients with hormone receptor-positive/human epidermal growth factor receptor 2-negative (HR+/HER2−) metastatic breast cancer (mBC), demonstrating improved survival outcomes compared to ET alone [14,15,16,17,18,19,20]. Studies comparing CDK4/6 inhibitors with systemic chemotherapy in patients with HR+/HER2− mBC have yielded confusing results [21,22,23,24,25,26]. Guidelines advocate for systemic chemotherapy in patients exhibiting rapid progression, symptoms, or visceral crises associated with HR+/HER2− mBC [27,28,29].

In our multicenter study, we aimed to investigate the impact of preferred first-line treatment modalities (systemic chemotherapy or CDK4/6 inhibitor combined with endocrine therapy) on progression-free survival and overall survival (OS) in patients with liver metastatic HR+/HER2− breast cancer as well as the prognostic and predictive influence of clinicopathological variables on survival outcomes.

## 2. Materials and Methods

### 2.1. Study Design

This multicenter, retrospective study included 121 patients with HR-positive/HER2−negative liver metastatic breast cancer diagnosed between May 2016 and November 2023, who had not previously received systemic chemotherapy or CDK4/6 inhibitor therapy for their advanced disease.

Inclusion criteria were as follows: histopathological confirmation of breast cancer; radiological evidence of metastatic disease; assessment of estrogen receptor (ER), progesterone receptor (PgR), and HER2 status by immunohistochemistry (IHC); patients who received at least one cycle of treatment (chemotherapy or CDK4/6 inhibitor) for liver metastatic disease; and radiological assessment of treatment response. Patients who had advanced-stage disease with locoregional recurrence or isolated bone metastases (without visceral metastases) before liver metastasis and were receiving only endocrine therapy at this stage were also included in the study. Exclusion criteria included incomplete radiological assessment of treatment response, insufficient clinical documentation, and unclear clinical outcomes.

### 2.2. Patient Population

We obtained clinicopathological data from databases and medical records. Data were collected on tumor histopathological features, de novo/recurrent metastatic disease status, patients’ age at liver metastatic stage (LMS), Eastern Cooperative Oncology Group (ECOG) performance status, endocrine-sensitive/resistant status of the disease, number and size of liver metastases, and whether local treatments were administered for liver metastases. The status of endocrine resistance was established in accordance with the 5th ESO-ESMO International Consensus Guidelines for Advanced Breast Cancer [30]. Patients with de novo metastatic disease or recurrence occurring more than 12 months after the completion of adjuvant therapy were categorized as endocrine-sensitive. Patients who experienced relapse during adjuvant therapy or within the first 12 months following the end of adjuvant therapy were categorized as endocrine resistant. We performed the assessments for ER, PgR, and HER2 status using available biopsy results from the primary tumor or metastatic lesion. Evaluation of ER, PgR, and HER2 status in patients was performed in accordance with the American Society of Clinical Oncology/College of American Pathologists (ASCO/CAP) guidelines [31,32]. In accordance with the St. Gallen consensus recommendations [33], we determined the threshold for Ki-67 and PgR expression levels at 20%, and we categorized patients as Ki-67-low (<20%) and Ki-67-high (≥20%); PgR-low (<20%) and PgR-high (≥20%). Patients with a HER2 IHC score of 0 were categorized as HER2−negative, while patients with a HER2 IHC score of +1, +2, and no gene amplification by in situ hybridization (ISH) were categorized as HER2−low [34].

### 2.3. Efficacy Outcomes

The time from the start of treatment to disease progression or death from any cause, whichever occurred first, was defined as progression-free survival (PFS). Patients who did not experience progression were censored at their last follow-up visit. Overall survival (OS) is defined as the duration from the initiation of systemic therapy to death from any cause or the last recorded visit. Patients who remained alive were censored from the date of analysis.

### 2.4. Ethical Considerations

The study adhered to the principles of the Declaration of Helsinki and received approval from the local clinical research ethics committee (date: 30 April 2024; number: E 83045809-604.01-985223). The retrospective design of this study obviated the requirement for informed consent.

### 2.5. Statistical Analysis

SPSS version 27 was used to perform statistical analysis. First, normal distribution was tested for quantitative variables. Variables that exhibited a normal distribution were analyzed using a t-test between the two groups. If the variables did not meet this criterion, they were compared using the Mann–Whitney U non-parametric test. The baseline categorical characteristics of the patients were compared using either Fisher’s exact test or the chi-squared test. The Kaplan–Meier method was used to estimate survival curves, which were compared using the log-rank test. Multivariate analysis was carried out using the Cox proportional hazards model to assess the effect of prognostic factors on PFS and OS. Hazard ratios (HR) with 95% confidence intervals (CI) were also calculated. Statistical significance was defined as a *p*-value of less than 0.05.

## 3. Results

### 3.1. Baseline Characteristics of Patients

The study group consisted of 121 patients. All patients were female. The median age at the liver metastatic stage was 53 years (range: 31–86). The majority of patients (71.1%) had invasive ductal carcinoma as the histological subtype of breast cancer. At the liver metastatic stage, 46 (38%) patients were premenopausal, whereas 75 (62%) patients were postmenopausal. ER expression was greater than 10% in all cases. The majority of patients (59.5%) presented with recurrent disease. There were fifteen patients who had isolated bone metastases or locoregional recurrences without visceral metastases at their first recurrence and received only antihormonal therapy (no systemic chemotherapy and no CDK4/6 inhibitor therapy). At the time of liver metastasis, 61 patients (51.4%) had hormone-resistant disease, while 60 patients (48.6%) had hormone-sensitive disease. In 69 patients (57%), the number of liver metastatic lesions was ≥4 lesions, with a median lesion size of 24 mm (range: 4–85 mm). Fourteen patients (11.6%) received local treatment modalities for liver metastases. Regarding systemic treatment at this stage, 44 patients (36.4%) initiated chemotherapy, whereas 77 patients (63.6%) were treated with CDK4/6 inhibitors in combination with endocrine therapy. Baseline characteristics of the study population are summarized in Table 1.

A statistically significant difference was observed between the chemotherapy and CDK4/6 inhibitor groups regarding the use of local treatment for liver metastases (20.5% in the chemotherapy group vs. 6.5% in the CDK4/6 inhibitor group; *p* = 0.021). In the liver metastatic stage, there was no statistically significant difference between the two groups regarding age, pre-treatment CA 15-3 levels, largest liver lesion size, metastatic disease status (de novo vs. recurrent), menopausal status (pre- vs. post-menopausal), ECOG performance score (0–1 vs. 2), hormonal status (endocrine sensitive vs. resistant), number of liver metastatic lesions (1–3 lesions vs. ≥4 lesions), PgR status (<20% vs. ≥20%), Ki67 status (<20% vs. ≥20%), and HER-2 status (zero vs. low). The comparison of clinical and tumor characteristics between the chemotherapy and CDK4/6 inhibitor groups in patients with liver metastases is outlined in Table 2.

Among the 44 patients who initiated chemotherapy, the most commonly used agents were paclitaxel (n = 21, 47%) and capecitabine (n = 6, 14%). Of the 77 patients who began treatment with a CDK4/6 inhibitor in combination with endocrine therapy, 41 (53.2%) received ribociclib and 36 (46.8%) received palbociclib.

In the initial radiological response assessment, objective responses were observed in 32 (72.7%) patients in the chemotherapy group and 44 (57.1%) patients in the CDK4/6 plus endocrine therapy group. There was no statistical difference in terms of radiological responses between the two groups (*p*: 0.088). Of the 77 patients who initiated treatment with CDK4/6 inhibitors, sixteen had no disease progression up to the data cutoff time. Among the 44 patients who started chemotherapy, 16 were switched to antihormonal therapy or CDK4/6 inhibitors based on a favorable disease response observed at the first radiological assessment. Treatment was discontinued in one patient in each group due to treatment-related toxicity. Patients received a median of three lines of treatment (min. 1–max. 11). Notably, 43 (98%) of the patients who initially received chemotherapy later received CDK4/6 inhibitors as part of their treatment regimen. Eleven patients (14%) who initiated treatment with CDK4/6 inhibitors did not receive post-CDK4/6 inhibitor treatment. Data on subsequent treatments are in Table 3.

**Table 1:** Baseline Characteristics of Patients.

		All Patients	
		(n: 121)	
**Initial Treatment in LMS, n (%)**	Chemoterapy	44	36.4
	CDK4/6 inhibitors plus ET	77	63.6
**Age (years)**	Median (Min.–Max.)	53	31–86
**Initial treatment in the metastatic stage, n (%)**	ET+/-OFS+/-Bisphosphonate/Denosumab	15	12.4
	Chemotherapy	38	31.4
	CDK4/6 inhibitors plus ET	68	56.2
**Histopathology, n (%)**	IDC	86	71.1
	ILC	9	7.4
	Other	26	21.5
**PgR Level, n (%)**	<%20	48	39.7
	≥%20	73	60.3
**Ki67, n (%)**	<%20	28	23.1
	≥%20	93	76.9
**HER-2 Status, n (%)**	Zero	81	66.9
	Low	40	33.1
**Metastatic Disease Status, n (%)**	De novo	49	40.5
	Recurrent	72	59.5
**Menopausal Status, n (%)**	Premenopausal	46	38.0
	Postmenopausal	75	62.0
**Hormonal Status, n (%)**	Endocrine sensitive	60	49.6
	Endocrine resistant	61	50.4
**ECOG, n (%)**	0–1	114	94.2
	2	7	5.8
**Number of LM Lesions, n (%)**	1–3 lesions	52	43.0
	≥4 lesions	69	57.0
**Largest LM Lesion Size (mm)**	Median (Min.–Max.)	24	5–85
**Local Treatment for the LM, n (%)**	No	107	88.4
	Yes	14	11.6
**Local Treatment Type, n (%)**	Resection	1	7.1
	RF Ablation	12	85.7
	TACE	1	7.1
**Pre-Treatment CA 15-3**	Median (Min.–Max.)	63.8	3–2957

CDK: cyclin-dependent kinase, ET: endocrine therapy, OFS: ovarian suppression therapy, IDC: invasive ductal carcinoma, ILC: invasive lobular carcinoma, LMS: liver metastatic stage, PgR: progesterone receptor, HER: human epidermal growth factor receptor, ECOG: Eastern Cooperative Oncology Group, LM: liver metastasis, RF: radiofrequency ablation, TACE: transarterial chemoembolization.

**Table 2:** Comparison of Clinical and Tumor Characteristics Between Chemotherapy and CDK4/6 Inhibitor Groups in Patients with Liver Metastases.

		Chemotherapy (n: 44)		CDK4/6 Inhibitors Plus ET (n: 77)		*p*
**Age**	Medyan (Min.–Max.)	50	31–85	54	31–86	0.126 ^t^
**Largest LM Lesion Size (mm)**	Medyan (Min.–Max.)	25	6–65	23	5–85	0.998 ^m^
**Pre-Treatment CA 15-3**	Medyan (Min.–Max.)	53	3–1976	80.3	4–2597	0.086 ^m^
**ECOG, n (%)**	0–1	42	95.5	72	93.5	0.659 *x*^2^
	2	2	4.5	5	6.5	
**Metastatic Disease Status, n (%)**	De novo	20	45.5	29	37.7	0.401 *x*^2^
	Recurent	24	54.5	48	62.3	
**Menopausal Status, n (%)**	Premenopausal	19	43.2	27	35.1	0.376 *x*^2^
	Postmenopausal	25	56.8	50	64.9	
**Hormonal Status, n (%)**	Endocrine sensitive	23	52.3	37	48.1	0.655 *x*^2^
	Endocrine resistant	21	47.7	40	51.9	
**HER-2 Status, n (%)**	Zero	33	75.0	48	62.3	0.154 *x*^2^
	Low	11	25.0	29	37.7	
**PgR Level, n (%)**	<%20	20	45.5	28	36.4	0.325 *x^2^*
	≥%20	24	54.5	49	63.6	
**Ki67, n (%)**	<%20	9	20.5	19	24.7	0.596 *x*^2^
	≥%20	35	79.5	58	75.3	
**Number of LM Lesions, n (%)**	1–3 lesions	15	34.1	37	48.1	0.136 *x*^2^
	≥4 lesions	29	65.9	40	51.9	
**Local Treatment for the LM, n (%)**	No	35	79.5	72	93.5	***0.021*** *x* ^2^
	Yes	9	20.5	5	6.5	
**Initial radiological response assessment**	Stabil or Progrese Disease	12	27.3	32	41.6	0.116 *x*^2^
	Objective Response	32	72.7	45	58.4	

*x*^2^*: Chi-square test, t: Student’s t-Test, m: Mann–Whitney U Test*. CDK: cyclin-dependent kinase, LM: liver metastasis, ECOG: Eastern Cooperative Oncology Group, HER: human epidermal growth factor receptor, PgR: progesterone receptor.

**Table 3:** Summary of All Lines of Subsequent Antineoplastic Medications by Drug Type.

	Chemotherapy	CDK4/6 Inhibitor
Patients who discontinued the first-line treatment, n	44	61
Any medication—no. (%)	44 (100.0)	50 (81.9)
Chemotherapy		
Anthraciycline+Cyclophosphamide	2	4
Platinum plus Taxane	2	5
Platinum plus Gemcitabine	9	14
Gemcitabine	5	6
Capecitabine	21	22
Paklitaxel/weekly	18	15
Ixabepilone	2	1
Vinorelbine	6	4
Other (Docetaxel, Cisplatin alone, Gemcitabine plus Capecitabine)	2	4
Hormone therapy		
Tamoxifen	1	1
Anastrozole	1	0
Letrozole	3	0
Exemestane	2	2
Fulvestrant	5	5
CDK4/6 inhibitor therapy		
Palbociclib plus Letrozole	4	0
Palbociclib plus Fulvestrant	6	0
Ribociclib plus Letrozole	16	0
Ribociclib plus Fulvestrant	17	0
Other		
Everolimus plus Exemestane	15	23
Alpelisib plus Fulvestrant	0	2

**Figure 1:**
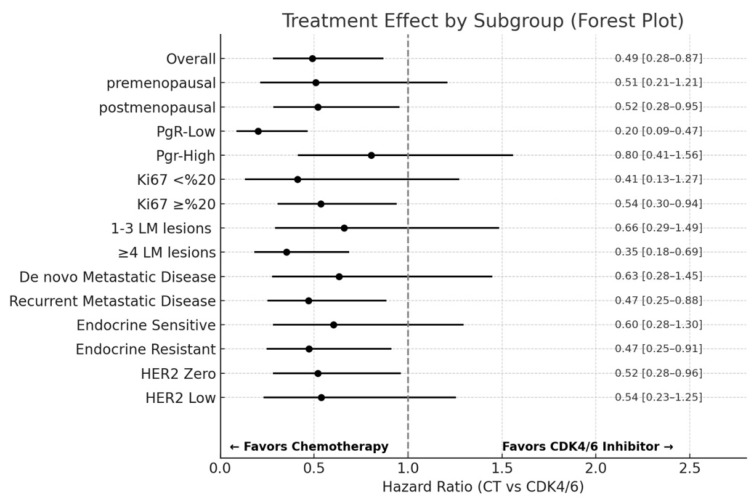
Subgroup Analyses of Survival Outcomes.

Subgroup analyses of survival outcomes demonstrated a statistically significant improvement in favor of chemotherapy over CDK4/6 inhibitors in the following categories; postmenopausal patients (HR: 0.52; 95% CI: 0.28–0.95), PgR-low tumors (HR: 0.20; 95% CI: 0.09–0.47), patients with Ki67 ≥20% (HR: 0.54; 95% CI: 0.30–0.94), patients with ≥4 liver metastases (HR: 0.35; 95% CI: 0.18–0.69), recurrent metastatic disease (HR: 0.47; 95% CI: 0.25–0.88), endocrine-resistant disease (HR: 0.47; 95% CI: 0.25–0.91), and HER2−zero tumors (HR: 0.52; 95% CI: 0.28–0.96). (Figure 1).

### 3.2. Survival Outcomes

The median follow-up time was 28.3 months (range: 1.7–99.8). The last follow-up date was 4 March 2025. By this date, 80 patients (66.1%) had died. The median overall survival for the total study population was 27.8 months (range: 1.3–99.7 months).

**Figure 2:**
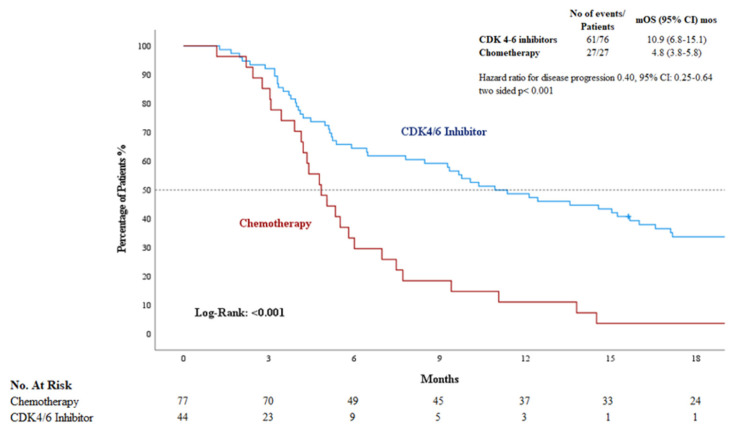
Kaplan–Meier curves of PFS in patients with Chemotherapy and CDK4/6 inhibitors. CDK: cyclin-dependent kinase; PFS: progression-free survival; mo: months.

**Figure 3:**
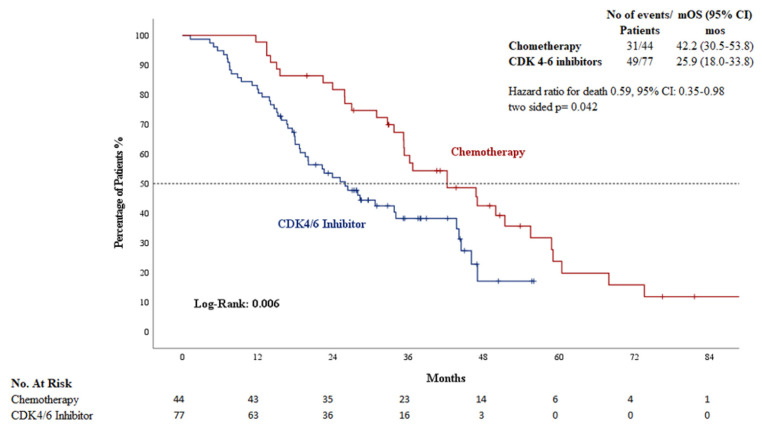
Kaplan–Meier curves of OS in patients with Chemotherapy and CDK4/6 inhibitors. CDK: cyclin-dependent kinase; OS: overall survival; mo: months.

The median progression-free survival was statistically longer in patients who initiated CDK4/6 inhibitors during the liver metastasis compared to those treated with chemotherapy (10.9 months [95% CI: 6.8–15.0] vs. 4.8 months [95% CI: 3.8–5.9]; *p* < ***0.01***) (Figure 2). The median overall survival was statistically longer in patients who initiated chemotherapy during the liver metastatic phase compared to those treated with CDK4/6 inhibitors (42.2 months [95% CI: 30.5–53.8] vs. 25.9 months [95% CI: 18.0–33.8]; *p = **0.042***) (Figure 3).

Univariate analyses revealed that several clinical and pathological factors were significantly associated with overall survival. During the liver metastatic period, poor overall survival was associated with the initiation of treatment using CDK4/6 inhibitors (*p* = ***0.007***), hormone-resistant disease status (*p = **0.010***), larger liver metastatic lesions (*p < **0.001***), and higher Ki67 levels (*p* = ***0.021***). Conversely, the administration of local liver interventions was significantly associated with improved survival (*p* = ***0.007***). In multivariate Cox regression analysis, the preferred first-line treatment during the liver metastatic phase (HR: 0.59, 95% CI: 0.35–0.98; *p* = ***0.042***), the size of the largest liver metastatic lesion (HR: 1.02, 95% CI: 1.00–1.04; *p* = ***0.002***), the hormonal status (HR: 1.71, 95% CI: 1.07–2.75; *p* = ***0.026***), and the application of liver-directed local therapy (HR: 0.32, 95% CI: 0.11–0.88; *p* = ***0.028***) were identified as independent prognostic factors for overall survival (Table 4).

There was no statistical difference in the use of chemotherapy or CDK4/6 inhibitors between primary and secondary endocrine refractory patients (*p* = 0.611), and no statistically significant difference in overall survival was observed between the two groups (mOS: 23.9 mo [95% CI: 18.8–29.1] vs. 27.9 mo [95% CI: 20.0–31.6]; *p* = 0.093).

## 4. Discussion

In our study, we evaluated the clinical outcomes of patients with HR+/HER2− metastatic breast cancer presenting with liver metastasis, focusing specifically on the impact of the first-line systemic treatment modality on overall survival. Our findings revealed that initiation of chemotherapy was associated with a statistically significant improvement in overall survival compared to CDK4/6 inhibitor-based endocrine therapy in this population. Subgroup analyses revealed that the survival advantage of chemotherapy was stronger in patients with endocrine-resistant disease, recurrent metastatic disease, postmenopausal status, multiple hepatic metastases (≥4 lesions), low progesterone receptor expression, higher Ki67 proliferation index, and HER2−zero malignancies. Furthermore, the use of local liver-directed therapies was correlated with prolonged survival, whereas larger liver metastatic lesion size and endocrine-resistant disease were independently related to inferior survival outcomes.

CDK4/6 inhibitors have demonstrated superior outcomes in progression-free survival [14,15,16,17,18,19,20] and overall survival [35,36,37,38,39] relative to endocrine treatments in phase 3 trials. These studies also included a subgroup analysis of patients with visceral metastatic disease. Subgroup analyses from the MONALEESA trials suggested a trend toward improved overall survival with the addition of ribociclib to endocrine therapy in patients with visceral metastatic disease; however, these findings did not reach statistical significance. Notably, in the liver metastatic subgroup of the MONALEESA-2 study, median overall survival was comparable between the ribociclib plus endocrine therapy arm (37.7 months) and the placebo plus endocrine therapy arm (38.1 months) [40].

Importantly, crossover to CDK4/6 inhibition following disease progression was not permitted in this study. In the MONALEESA-3 and MONALEESA-7 studies, approximately 60% of patients had endocrine-sensitive disease, and 25% of patients in the placebo group subsequently received a CDK4/6 inhibitor. And in both groups, the rate of subsequent chemotherapy use was around 30%, which can be considered an important factor in affecting survival times, especially in the placebo group. Abemaciclib showed a meaningful improvement in overall survival in the MONARCH 2 and 3 studies, even for patients with visceral metastases. Palbociclib did not improve overall survival in the PALOMA trials.

Previous studies comparing endocrine therapies to chemotherapy indicated that approximately 25% of patients with HR+/HER2− metastatic breast cancer initiated treatment with chemotherapy, which yielded inferior outcomes relative to endocrine therapy, according to research conducted by the Southwest Netherlands Breast Cancer Consortium from 2007 to 2009 [41]. In this study, patients who initiated chemotherapy had particularly aggressive tumor characteristics and visceral metastases, which could have created a bias for chemotherapy preference and been reflected in the results. In a study conducted on a population selected by propensity score matching, it was shown that there was no difference in survival between chemotherapy and endocrine treatments [23]. A meta-analysis of randomized studies from 2000 to 2016, conducted after the initial publications of phase 3 trials involving CDK4/6 inhibitors, demonstrated that combinations of palbociclib with letrozole or fulvestrant were superior to several chemotherapy regimens regarding progression-free survival in postmenopausal patients [42]. Similarly, a systematic review and meta-analysis including phase 2 and 3 randomized controlled trials with ET or CT published from 2000 to 2017 indicated that chemotherapeutic regimens were not superior to CDK4/6 inhibitor combinations regarding progression-free survival [22]. Both meta-analyses encompassed a broad time span of 16 to 17 years. Some of the chemotherapy studies had limitations due to heterogeneity in the patient population, dosing in applied treatments, and evaluation criteria, such as the inclusion of patients with triple-negative metastatic breast cancer in some of the studies. Neither meta-analysis provided data on the visceral metastatic subgroup, nor did they present information on overall survival.

In the phase 2 KENDO trial, CDK4/6 inhibitors seemed to provide longer progression-free survival (PFS) and overall survival (OS) than chemotherapy for patients with HR+/HER2− advanced breast cancer, but this was only noticeable in the Luminal A group. Approximately 40% to 45% of patients in the entire cohort had visceral metastases. Patients who responded to chemotherapy prior to disease progression in the chemotherapy arm were eligible for maintenance therapy with endocrine therapy. However, with early closure and only 49 patients in total, the data lacked sufficient statistical power to demonstrate a superiority difference between the treatment arms [25].

The phase 3 PEARL trial, which investigated treatment options in postmenopausal patients with aromatase inhibitor (AI)-resistant HR-positive/HER2−negative breast cancer, found that palbociclib in combination with exemestane or fulvestrant failed to demonstrate statistical superiority over capecitabine in AI-resistant disease independent of ESR-1 mutation. On the other hand, capecitabine demonstrated superior efficacy over palbociclib in combination with exemestane or fulvestrant in the non-luminal group. Additionally, there was a numerical difference in PFS in favor of capecitabine in the ESR-1 mutant group. Notably, 40% to 43% of patients in the entire cohort had hepatic metastatic disease [24]. Consistent with these findings, our study found that initiating treatment with chemotherapy was significantly more effective in terms of overall survival in postmenopausal patients and AI-resistant disease.

In the phase 2 YOUNG PEARL study in premenopausal patients, there was no difference in overall survival between palbociclib plus endocrine therapy and capecitabine [26]. Palbociclib did not show an OS benefit in the PALOMA 2 and 3 studies, but the overall survival in this study was similar to that in the ribociclib and abemaciclib studies. The post-progression CDK4/6 inhibitor use rates in this study were also higher than in other phase 3 studies. In our study, almost all patients in the chemotherapy group received CDK4/6 inhibitors in their subsequent treatments after treatment discontinuation. The result may have been reflected in the survival time.

The phase 2 RIGHT CHOICE trial reported superior progression-free survival with ribociclib-based combinations compared to chemotherapy [21]. Although a benefit in progression-free survival was also noted among patients with liver metastases, the difference did not reach statistical significance and was not superior to treatment in recurrent metastatic disease. At the 30-month follow-up, overall survival was comparable between the treatment arms (66.6% vs. 64.6%). Notably, the trial excluded postmenopausal patients; 65% of the study population had de novo metastatic disease, and the majority had endocrine-sensitive disease. The patients included in the RIGHT CHOICE study were rich in patient characteristics that showed CDK4/6 inhibitor treatment to be more effective when we evaluated the subgroup analyses of the MONALEESA studies. In our study, approximately 62% of the patients were postmenopausal, 50% had endocrine resistance, and 60% had recurrent disease.

Approximately 60% of patients with HR+/HER2− breast cancer present with endocrine-resistant disease following completion of definitive therapy [11]. Evidence supporting the use of CDK4/6 inhibitors in the endocrine-resistant population remains limited due to the lack of robust data. The MONALEESA-2 (in which 98% of patients were endocrine-sensitive) and MONARCH-3 (which enrolled only endocrine-sensitive patients) trials included exclusively endocrine-sensitive populations [35,38]. The only phase 3 trial to enroll exclusively endocrine-resistant patients and to stratify them by primary or secondary endocrine resistance, MONARCH-2, demonstrated both clinically and statistically significant overall survival benefits with abemaciclib [39]. However, due to the small sample size, the survival benefit observed in the primary resistance subgroup did not reach statistical significance. In contrast, other trials that included endocrine-resistant populations, such as MONALEESA-3, MONALEESA-7, and PALOMA-3, did not demonstrate a clinically meaningful overall survival benefit with the addition of CDK4/6 inhibitors to endocrine therapy [43,44,45]. Particularly, primary endocrine-resistant disease tends to present more frequently with visceral metastases, especially hepatic involvement, and represents the subgroup with the poorest prognosis compared to secondary endocrine-resistant or endocrine-sensitive disease [11]. Considering that the benefit derived from chemotherapy diminishes as its use is delayed in metastatic HR+/HER2− breast cancer [46], chemotherapy may be preferred over CDK4/6 inhibitors as a first-line treatment option in selected patients.

In patients with metastatic HR+/HER2− breast cancer treated with CDK4/6 inhibitors, a high Ki67 index and low PgR expression were associated with shorter PFS and OS [10,13]. In our study, in multivariate analyses using Cox regression, Ki67 levels (<20% vs. ≥20%) and PgR expression levels (<20% vs. ≥20%) were not independent factors on their own, independent of treatment, although it should not be ignored that we reached this conclusion with a small number of patients. In subgroup analyses, the survival advantage with chemotherapy was more pronounced in these subgroups.

When we interpret the study suggesting that HER2−low tumors have lower pathological complete response rates than HER2−zero tumors in hormone-positive tumors, we can say that HER2−low tumors respond worse to chemotherapy [12]. However, such treatment did not seem to have an effect on overall survival [47]. In the PALOMA 2 and studies, the benefit of palbociclib in the HER2−zero group was limited [48]. Although there are retrospective data suggesting that there is no difference between HER2−low and zero [49], a meta-analysis found a higher risk of death in HER2−low patients [50]. In our study, HER2 status was not an independent variable for overall survival in the multivariate Cox regression analysis, independent of the treatment arms. However, the effectiveness of chemotherapy in terms of overall survival was more pronounced in the HER2−zero subgroup.

Information on the dimensions of liver metastases is primarily derived from studies involving patients with colorectal cancer. A negative correlation has been observed between the size of liver metastatic lesions and survival outcomes [51,52,53]. Our study found that larger liver metastatic lesions were associated with poorer survival results.

Particularly in patients with de novo metastatic breast cancer, both loco-regional treatment and metastasis-specific local treatment have the potential to prolong survival, although current data are limited [54,55,56,57]. There were studies suggesting that combining systemic therapy with ablation in patients with liver metastatic breast cancer had a survival benefit, even in the presence of extrahepatic metastases [58]. Ablation appeared to be a reliable method that could contribute to survival in selected patients [59,60]. Although it was applied to a small number of patients in our study, the overall survival outcomes were superior in patients who underwent local treatment. Despite a limited number of patients undergoing local treatment, the statistically significant difference in the application of local treatment between the two groups may have influenced the survival outcomes.

ESR1 mutations are infrequently observed in initial breast cancer [61], but are more prevalent in breast cancer patients who have undergone prior treatment with aromatase inhibitors, particularly in advanced stages [62]. A study assessing ESR1 mutations in available archived baseline plasma from phase 3 trial Faslodex to Exemestane with or without Arimidex (SoFEA) and the PALOMA 3 trials found that ESR1 mutations correlated with acquired resistance to aromatase inhibitors. In this study, fulvestrant alona was superior to exemestane, and the palbociclib plus fulvestrant combination was superior to fulvestrant alone in terms of progression-free survival in ESR1 mutant patients. This study did not provide overall survival data [63]. In one study providing real-world data, ESR1 mutant and ESR1 wild-type patients treated with CDK4/6 inhibitors had similar real-world survival, but ESR1 mutant patients receiving AI had worse outcomes, whereas those receiving fulvestrant had comparable outcomes [64].

Mutations in PIK3CA, seen in approximately 40% of patients with HR+/HER2− metastatic breast cancer, can induce endocrine resistance through the activation of the PIK3 pathway and are associated with an unfavorable prognosis [61,65]. Patients with HR+/HER2− metastatic breast cancer with PIK3CA mutations are less sensitive to chemotherapy [65]. Conversely, CDK4/6 inhibitors have demonstrated effectiveness irrespective of PIK3CA mutations in phase 3 trials [14,66,67].

Although PIK3CA and ESR1 are known drivers of tumor growth, further studies are needed to understand whether these mutations are responsible for resistance to the combination of CDK4/6 inhibitors plus ET.

Retrospective studies of patient samples from PALOMA 2 and 3 trials showed that the benefit observed in non-luminal subtypes was less than in luminal subtypes [68,69]. A retrospective analysis of tumor samples from the MONALEESA trials showed that patients with only basal-like disease had poor responses to endocrine therapy, with or without ribociclib, while the HER2−enriched subtype demonstrated the greatest benefits for progression-free survival and overall survival. Considering the limited sample size of nın-luminal patients and the fact that these data were derived from subgroup analyses, it is evident that additional research is required to inform treatment decisions for individuals exhibiting non-luminal characteristics [70].

Consider several limitations when interpreting these real-world data results. First, retrospective acquisition of data from clinical databases may introduce potential selection biases and factors that may influence the interpretation of results. Second, this study lacked information on intrinsic subtypes and other molecular features (ESR1 mutation, PIK3CA, and AKT signaling pathway alterations) that could potentially affect prognosis or response to treatment. In our study, eleven patients who underwent CDK4/6 inhibitors as first-line therapy were unable to receive further treatment due to disease progression or mortality. Furthermore, nearly all patients in the chemotherapy group underwent additional CDK4/6 inhibitor therapies. These conditions may have influenced the overall survival outcomes. Another limitation is that we were unable to include patients receiving abemaciclib in our study because it is not covered by insurance in our country. The small sample size—especially the smaller chemotherapy group—posed a difficulty, even though we first thought of using propensity score matching (PSM) to lower any treatment allocation bias. Matching would have caused significant data loss and lower statistical power, perhaps restricting the interpretability and generalizability of our results. In multivariate models, we thus decided to show the complete cohort analysis together with controlling for pertinent factors. We underline the requirement of larger prospective datasets to enable matched comparison analyses in the future, since we admit that the lack of PSM is a methodological restriction.

## 5. Conclusions

Patients with HR-positive/HER-2−negative metastatic breast cancer involving the liver represent a clinically challenging subgroup characterized by aggressive disease biology and poor survival outcomes. In this context, the selection of first-line systemic therapy assumes heightened clinical relevance. Although CDK4/6 inhibitors are still the mainstay of HR+/HER2− mBC treatment, our results imply that some patients with unfavorable biological profiles (low PR, high Ki67, and HER2−zero), multiple liver metastatic, endocrine-refractory, and recurrent disease would benefit more from chemotherapy as the first course of treatment. When liver metastatic disease is inherently predicted to have an unfavorable prognosis, treatment planning should incorporate other adverse prognostic biomarkers. Additionally, a thorough evaluation of local liver therapy for select patients with HR+/HER2− breast cancer that has metastasized to the liver appears justified. Additional prospective studies are needed to confirm the effect of this preference on survival outcomes.

## Figures and Tables

**Table 4:** Univariate and Multivariate Cox Regression Analyses for Overall Survival.

	Univariate			Multivariate		
	HR	%95 CI	*p*	HR	%95 CI	*p*
Age	1.01	0.99–1.03	0.176			
Menopausal Status (pre vs. post)	1.52	0.95–2.42	0.078			
ECOG (0–1 vs. 2)	1.96	0.85–4.54	0.116			
Metastatic Disease Status (de novo vs. recurrent)	0.64	0.40–1.03	0.067			
Hormonal Status (endocrine sensitive vs. resistant)	1.80	1.15–2.83	***0.010***	1.71	1.07–2.75	***0.026***
ER Level	0.99	0.98–1.00	0.082			
PgR (<%20 vs. ≥%20)	0.69	0.44–1.07	0.096			
HER-2 Status (Zero vs. Low)	1.31	0.82–2.11	0.260			
Ki67	1.02	1.00–1.03	***0.021***			
Ki-67 (<%20 vs. ≥%20)	0.88	0.52–1.48	0.629			
Number of LM Lesions (1–3 vs. ≥4)	1.46	0.92–2.29	0.106			
Largest LM Lesion Size (mm)	1.02	1.00–1.04	***<0.001***	1.02	1.00–1.04	***0.002***
Local Treatment for the LM (no vs. yes)	0.28	0.11–0.71	***0.007***	0.32	0.11–0.88	***0.028***
Initial Treatment in LMS (CDK4/6 inh vs. ChT)	0.51	0.31–0.83	***0.007***	0.59	0.35–0.98	***0.042***

HR: hazard ratio, ECOG: Eastern Cooperative Oncology Group, ER: estrogen receptor, PgR: progesterone receptor, HER: human epidermal growth factor receptor, LM: liver metastasis, LMS: liver metastatic stage, ChT: chemotherapy, CDK4/6 inh.: cyclin-dependent kinase 4/6 inhibitors.

## Data Availability

The data presented in this study are available on request from the corresponding author.

## Abbreviations

AI	Aromatase inhibitor
ASCO	American Society of Clinical Oncology
CAP	College of American Pathologists
CDK4/6	Cyclin-dependent kinase 4/6
CI	Confidence interval
ChT	Chemotherapy
ECOG	Eastern Cooperative Oncology Group
ER	Estrogen receptor
ET	Endocrine therapy
HER2	Human epidermal growth factor receptor 2
HR	Hormone receptor
IDC	Invasive ductal carcinoma
IHC	Immunohistochemistry
ILC	Invasive lobular carcinoma
ISH	In situ hybridization
LM	Liver metastasis
LMS	Liver metastatic stage
OFS	Ovarian function suppression
OS	Overall survival
PFS	Progression-free survival
PgR	Progesterone receptor
RF	Radiofrequency
TACE	Transarterial chemoembolization
mBC	Metastatic breast cancer
